# Breeder age outweighs egg grading method on hatchability and chick quality in broiler hatcheries

**DOI:** 10.1007/s11250-026-05417-8

**Published:** 2026-10-18

**Authors:** Jumara Coelho Ticiano, Roberto Carlos de Freitas, Luzianna Macedo Fonseca, Dauisia Corsino de Souza, Robson Mateus Freitas Silveira, Lúcio Francelino de Araújo, Arele Arlindo Calderano

**Affiliations:** 1 Department of Animal Science, School of Animal Science and Food Engineering (FZEA), University of São Paulo (USP), Pirassununga, SP Brazil; 2 Grupo Alvorada, Itapetininga, SP Brazil; 3 School of Veterinary Medicine and Animal Science, São Paulo State University (UNESP), Botucatu, São Paulo Brasil; 4 Department of Animal Science, “Luiz de Queiroz” College of Agriculture (ESALQ), University of São Paulo (USP), Piracicaba, SP Brazil; 5 Department of Biosystems Engineering, “Luiz de Queiroz” College of Agriculture (ESALQ), University of São Paulo (USP), Piracicaba, SP 13418-900 Brazil; 6 Department of Animal Science, Federal University of Viçosa (UFV), Viçosa, MG Brazil

**Keywords:** Classification of fertile eggs, Production efficiency, Embryo diagnosis, Industrial incubation, Chick quality

## Abstract

Incubation efficiency and chick health are fundamentally linked to fertile egg quality, which is influenced by both grading methods and breeder age. This study evaluated the effects of manual versus automated grading and Cobb breeder age (28–42, 43–56, and 57–70 weeks) on hatchability, chick quality, and embryonic mortality. The study was conducted in a commercial hatchery and a total of 54,432 fertile eggs were incubated and distributed in a completely randomized design in a 2 × 3 factorial scheme (two sorting methods × three age groups). Egg quality was first assessed by identifying cracked, dirty, inverted, and rejected eggs. Post-incubation, the study evaluated hatchability and chick quality (categorized into first and second grades). A detailed embryo diagnosis was performed to determine the incidence of rejects, embryonic mortality by stage, and the presence of live or dead pecked eggs. Data were statistically analyzed to evaluate the influence of classification methods and breeder age on key production performance indicators. This study revealed that breeder age significantly affected hatchability and incubation performance. Younger breeder hens demonstrated higher hatchability rates, whereas eggs from older breeder hens exhibited reduced incubation efficiency. In contrast, automated and manual grading systems showed comparable hatchability performance, with no significant differences observed between classification methods. These findings indicate that automated grading may be applied without negatively affecting hatchability outcomes across different breeder age groups.

## Introduction

Poultry farming continues to solidify its role as a strategic agricultural activity, driving the economy and securing food supplies. The country occupies a prominent position, consolidating itself as the world’s largest exporter of chicken meat. In 2025, approximately 5.7 billion broilers were slaughtered, generating 15.3 million tons of chicken meat, the poultry production chain has a direct impact on the national economy, generating around 2 million direct and indirect jobs (IBGE 2022; Santos 2024; ABPA 2026).

This sector consists of interdependent stages, ranging from the production of fertile eggs to the rearing and slaughter of chickens, ensuring quality products for the consumer market (Vivan 2019). Within this process, the incubation phase stands out as critical, being fundamental for the supply of day-old chicks with high zootechnical performance, essential to meet the growing demand of the global market (Maia et al. 2023).

The efficiency of the incubation process is directly influenced by the quality of fertile eggs, with classification being a decisive step in the production chain (Nazareno et al., 2026). Proper categorization of eggs, considering factors such as weight, size, shell integrity, and cleanliness, is essential to ensure uniform hatching and the production of healthy chicks (Semeão et al. 2023). Effective egg classification is a critical component of poultry management, as selecting the best eggs for incubation significantly improves hatchability rates and optimizes operational efficiency. By identifying and removing abnormal, damaged, or infertile eggs before incubation, producers minimize waste and maximize the return on incubation space (Reijrink et al. 2009; Damaziak et al. 2021). In addition, egg quality is a critical factor in embryo health and development, directly impacting the sustainability and competitiveness of the poultry industry (Hincke et al. 2019; Vekic et al. 2021).

Among the factors that affect the quality of hatchable eggs, the age of the breeder hens stands out as a determining factor, directly influencing the quality of fertile eggs and, consequently, the success of the incubation process. Young breeder hens tend to produce smaller eggs with a higher proportion of albumen and more resistant shells, but with a lower yolk concentration, which can impact embryonic development and chick quality (Oliveira and Santos 2018; Schadeck 2020). On the other hand, older breeder hens produce larger eggs with a greater amount of yolk, suitable for embryonic growth, but with thinner and less resistant shells, increasing the risk of breakage and loss during incubation (Demirel and Demirel 2020; Gregrova et al. 2024). These variations are directly linked to physiological changes in broilers throughout their reproductive life, reinforcing the need for adjustments in egg management and classification based on the age of the breeder hens (Silveira et al., 2026).

Furthermore, the age of the breeder hens is a key driver of incubation success, specifically affecting hatch rates and overall chick quality at birth. Studies highlights that breeder hens within their initial production phase, particularly up to 42 weeks of age, achieve peak performance, as their eggs provide the ideal balance between shell strength and internal nutrient composition (Schadeck 2020; Gregrova et al. 2024). After this period, a gradual decrease in egg quality is observed, which can compromise chick health and zootechnical indicators (Reijrink et al. 2009; Silva et al. 2017). These data reinforce the importance of appropriate management strategies and the application of accurate classification techniques to mitigate the negative impacts associated with the advanced age of the breeder hens, ensuring the productive efficiency and sustainability of the poultry sector.

This study aimed to evaluate the effects of breeder age and egg classification method on hatchability, chick quality, and embryonic mortality in commercial broiler breeders. Additionally, the study investigated whether classification practices may influence incubation performance across different breeder age groups.

## Materials and methods

This study was conducted at a commercial hatchery in Itapetininga, São Paulo, Brazil (23°35′ S, 48°03′ W; altitude ~ 670 m). The local humid subtropical climate (Köppen-Geiger Cfa) features average annual temperatures of 22 °C (68–72 °F) and annual precipitation between 1,200 and 1,400 mm.

### Egg selection and classification

Fertile eggs from Cobb heavy breeder hens were categorized into three groups: young (28–42 weeks), intermediate (43–56 weeks), and old (57–70 weeks). To ensure superior shell quality, samples were prioritized from the day’s second collection and gathered manually from nests avoiding conveyor belts before immediate transport to the hatchery.

**Fig. 1:**
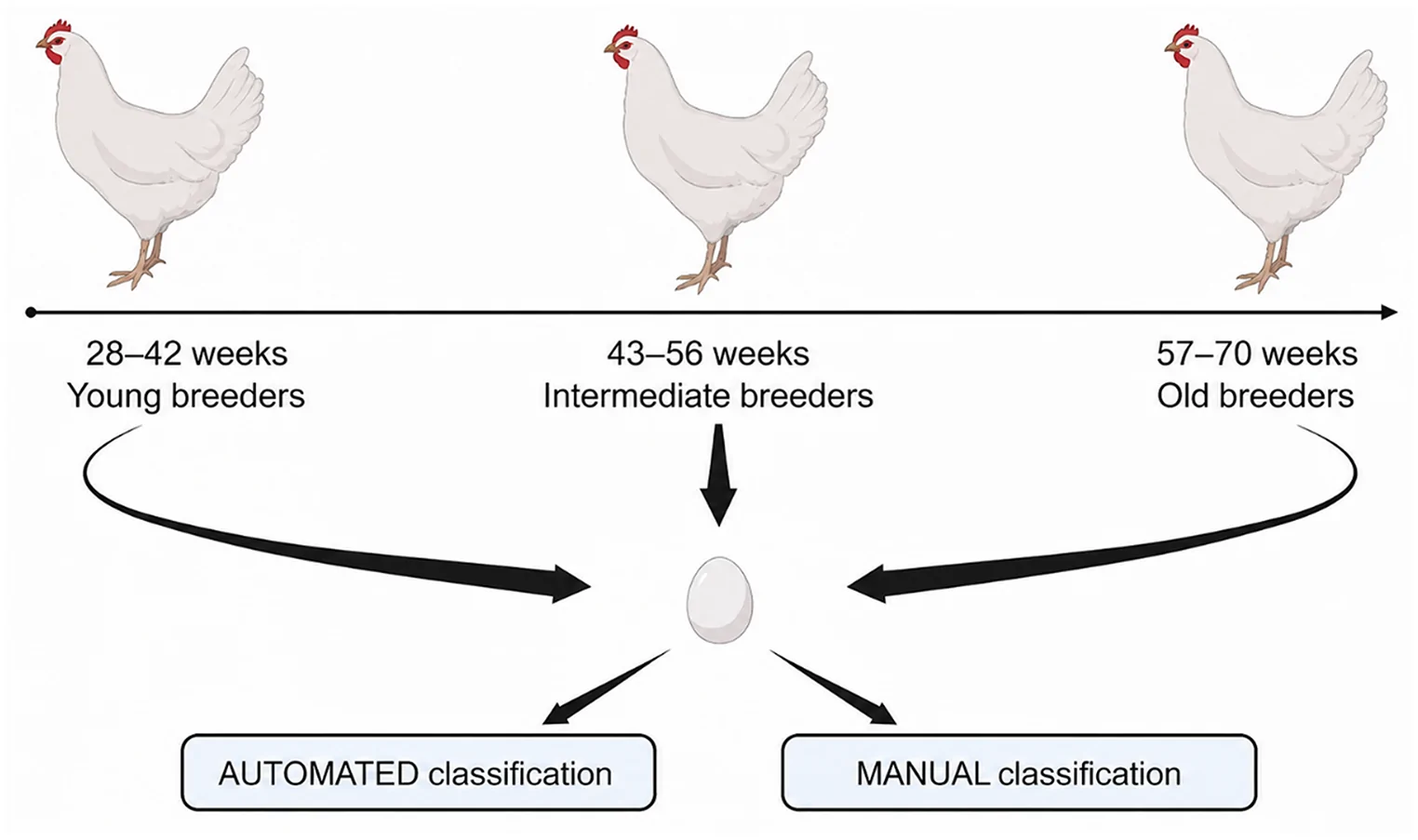
Experimental design I. Eggs from the same flock were evaluated throughout the productive life of the breeder hens, classified as young breeder hens (28–42 weeks), intermediate breeder hens (43–56 weeks) and old breeder hens (57–70 weeks). Six trays with a capacity of 84 eggs were subjected to each type of classification (manual and automated)

At the hatchery, eggs were divided equally between manual and automated classification methods for a direct comparison. Manual sorting was conducted by trained personnel using visual and tactile inspection to identify defects such as cracks, dirt, inverted positioning (small end up), and shell irregularities. In contrast, the automated system utilized high-precision optical sensors to detect these same quality parameters illustrated in Fig. 1.

Trays were color-coded and numbered by treatment, then positioned in the front of the incubators. To prevent temperature-related bias, the top and bottom two shelves were excluded. Across nine incubation cycles, tray placement was systematically rotated to ensure environmental uniformity and minimize position effects.

The hatchery operated using a single-stage system (Coopermaq, Brazil) under controlled environmental conditions. Incubation temperature was maintained at 37.5 °C ± 0.3 °C, while relative humidity ranged from 60% to 65% during incubation and from 70% to 80% during hatching, according to embryonic development stage and hatchery operational standards. Carbon dioxide (CO₂) levels were automatically maintained between 0.4% and 0.5%. Eggs were automatically turned hourly at angles ranging from 38º to 45º to ensure proper embryonic development and thermal uniformity. Ventilation and internal air circulation were automatically controlled to maintain environmental homogeneity within the incubators. To ensure spatial representativeness, trays were distributed across three internal incubator regions: near the wall, center, and fan area. All eggs were set within 24 h after grading, and transfer to hatchers occurred on day 19 of incubation according to the hatchery standard operating procedure.

Detailed technical data sheets were used to document each treatment, recording the flock number, breeder age, classification method, incubator ID, and dates for laying, incubation, transfer, and hatching.

This methodology ensured experimental rigor, traceability, and data reliability, providing a robust framework for analyzing how breeder age and classification methods impact reproductive indicators.

### Experimental design

The study followed a completely randomized design using a 2 × 3 factorial scheme, crossing two grading methods (manual and automated) with three breeder age groups: young (28–42 weeks), intermediate (43–56 weeks), and old (57–70 weeks); resulted in six distinct treatments. For each treatment, 12 experimental units per treatment per incubation were used, each unit consisting of a tray with 84 fertile eggs. The experiment was repeated over nine independent incubations in time (time blocks), total 648 experimental units (6 treatments × 12 repetitions × 9 blocks), which corresponds to 54,432 fertile eggs incubated throughout the study. To ensure experimental control, trays were systematically interleaved within the incubation carts to mitigate internal thermal and environmental variability.

### Evaluated variables

The study analyzed several critical factors to determine egg viability and incubation success:

Egg Classification: Eggs were assessed for defects including cracks, dirt, inverted positioning (small end up), and total rejections to determine suitability for incubation.

Hatch Rate: Calculated as the percentage of fertile eggs that produced viable chicks.

Chick Quality: Classified into three distinct tiers:

- First Category: Vigorous chicks with healed navels, bright eyes, hydrated legs, and uniform plumage.
- Second Category: Chicks with minor imperfections, such as poorly healed navels, tarsal lesions, or low vigor.
- Rejects: Chicks deemed unviable.

Embryonic Mortality: Determined via embryo diagnosis and categorized by stage: initial (0–7 days), intermediate (8–14 days), and final (15–21 days).

Hatch Failure: Analysis included live pecked eggs (embryos that began but did not finish hatching) and dead pecked eggs (failures in the final developmental stages).

### Statistical analyses

Data was analyzed using a Generalized Linear Model (GLM) with breeder age, classification method, and their interactions as predictor variables. An Identity link function with a Gaussian distribution was applied to analyze hatchability data. Statistical significance was considered at *p* < 0.05. Descriptive statistics for hatchability, chick quality, embryonic mortality, and egg classification variables according to breeder age, grading method, and their factorial combinations are presented in Tables 1 and 2.

**Table 1:** Descriptive statistics for egg quality defects and waste according to breeder age and grading method

Age group	Grading method	Inverted eggs	Cracked eggs	Dirty eggs	Rejects	Waste
Young	Automated	0.45 ± 0.68	0.23 ± 0.54	0.35 ± 0.73	0.30 ± 0.62	0.56 ± 0.83
Young	Manual	0.79 ± 0.90	0.16 ± 0.48	0.38 ± 0.61	0.28 ± 0.84	0.70 ± 0.89
Intermediate	Automated	0.67 ± 0.92	0.37 ± 0.66	0.51 ± 0.98	0.17 ± 0.54	0.59 ± 0.82
Intermediate	Manual	0.98 ± 1.09	0.07 ± 0.30	0.72 ± 1.17	0.31 ± 0.57	0.84 ± 1.10
Old	Automated	0.72 ± 0.96	0.50 ± 0.85	0.60 ± 1.49	0.25 ± 0.69	0.73 ± 0.92
Old	Manual	1.56 ± 1.31	0.19 ± 0.41	0.54 ± 1.13	0.58 ± 0.92	1.00 ± 1.10

Source: Adapted from Jamovi outputs generated from the experimental database

**Table 2:** Descriptive statistics for embryonic mortality and hatchability according to breeder age and grading method

Age group	Grading method	Mortality 0–7 d	Mortality 8–14 d	Mortality 15–18 d	Mortality 19–21 d	Hatchability
Young	Automated	3.06 ± 1.92	0.44 ± 0.67	0.72 ± 0.75	0.67 ± 0.84	88.91 ± 3.23
Young	Manual	3.14 ± 1.89	0.44 ± 0.69	0.71 ± 0.84	0.97 ± 0.95	88.58 ± 4.00
Intermediate	Automated	3.44 ± 1.91	0.46 ± 0.72	0.74 ± 0.78	0.99 ± 0.97	85.42 ± 4.18
Intermediate	Manual	3.01 ± 1.77	0.32 ± 0.59	0.61 ± 0.82	1.22 ± 1.25	86.28 ± 4.16
Old	Automated	3.62 ± 1.96	0.65 ± 0.82	0.83 ± 0.89	1.31 ± 1.08	83.08 ± 4.35
Old	Manual	3.80 ± 1.33	0.49 ± 0.70	0.80 ± 0.88	1.36 ± 1.49	83.26 ± 4.13

Source: Adapted from Jamovi outputs generated from the experimental database

## Results and discussion

The results are presented by first examining the individual effects of breeder age and classification method, followed by the interaction between breeder age and grading method. These results are summarized in Table 3. Breeder hens age showed a significant effect on hatchability rates (*p* < 0.0001), with younger flocks presenting higher hatchability compared to older breeder hens. In contrast, grading method and the interaction between breeder age and grading method did not significantly affect hatchability performance.

**Table 3:** Omnibus tests for fixed effects in the generalized linear model evaluating hatchability according to breeder age and grading method

Effect	χ²	df	*P* value	η²
Breeder age	207.543	2	< 0.0001	0.0150
Grading method	0.553	1	0.4571	0.00004
Breeder age × grading method	2.380	2	0.3043	0.00017

Source: Adapted from generalized linear model outputs generated in Jamovi from the experimental database

Table 4 presents the parameter estimates obtained from the generalized linear model using a Gaussian distribution with Identity link function. Breeder age significantly affected hatchability rates, with intermediate and old breeder hens showing lower hatchability compared to young breeders. In contrast, grading method and the interaction between breeder age and grading method did not significantly affect hatchability performance.

**Table 4:** Parameter estimates from the generalized linear model evaluating hatchability according to breeder age and grading method

Effect	Estimate	SE	95% confidence intervals	*P* value
Intercept	85.920	0.158	85.610 to 86.230	< 0.0001
Intermediate vs. Young	-2.899	0.387	-3.658 to -2.140	< 0.0001
Old vs. Young	-5.578	0.387	-6.337 to -4.819	< 0.0001
Manual vs. Automated	0.235	0.316	-0.385 to 0.855	0.4571
Intermediate × Manual	1.190	0.775	-0.328 to 2.709	0.1243
Old × Manual	0.507	0.775	-1.011 to 2.025	0.5127

Source: Adapted from generalized linear model outputs generated in Jamovi from the experimental database

Post hoc comparisons demonstrated progressive reductions in hatchability with advancing breeder age, as shown in Table 5. Young breeder hens showed significantly higher hatchability rates compared to both intermediate and old flocks, while intermediate breeders also presented higher hatchability than old breeder hens, confirming a consistent age-related decline in incubation performance.

**Table 5:** Post hoc comparisons among breeder age groups for hatchability using Bonferroni adjusted tests

Comparison	Mean difference	SE	z value	Bonferroni adjusted *P* value
Young vs. Intermediate	2.899	0.387	7.486	< 0.0001
Young vs. Old	5.578	0.387	14.403	< 0.0001
Intermediate vs. Old	2.679	0.387	6.917	< 0.0001

Source: Adapted from Jamovi outputs generated from the experimental database. P values < 0.05 were considered statistically significant

Post hoc comparisons for grading methods showed no statistically significant differences in hatchability rates between automated and manual classification systems, as presented in Table 6. Likewise, the interaction between breeder age and grading method did not significantly affect hatchability outcomes, indicating that the effects of breeder age occurred independently of the classification method used (Table 6).

**Table 6:** Post hoc comparison between grading methods for hatchability using Bonferroni adjusted tests

Comparison	Mean difference	SE	z value	Bonferroni adjusted *P* value
Automated vs. Manual	-0.235	0.316	-0.744	0.4573

Source: Adapted from generalized linear model outputs generated in Jamovi from the experimental database. Significant differences were considered at *p* < 0.05

**Fig. 2:**
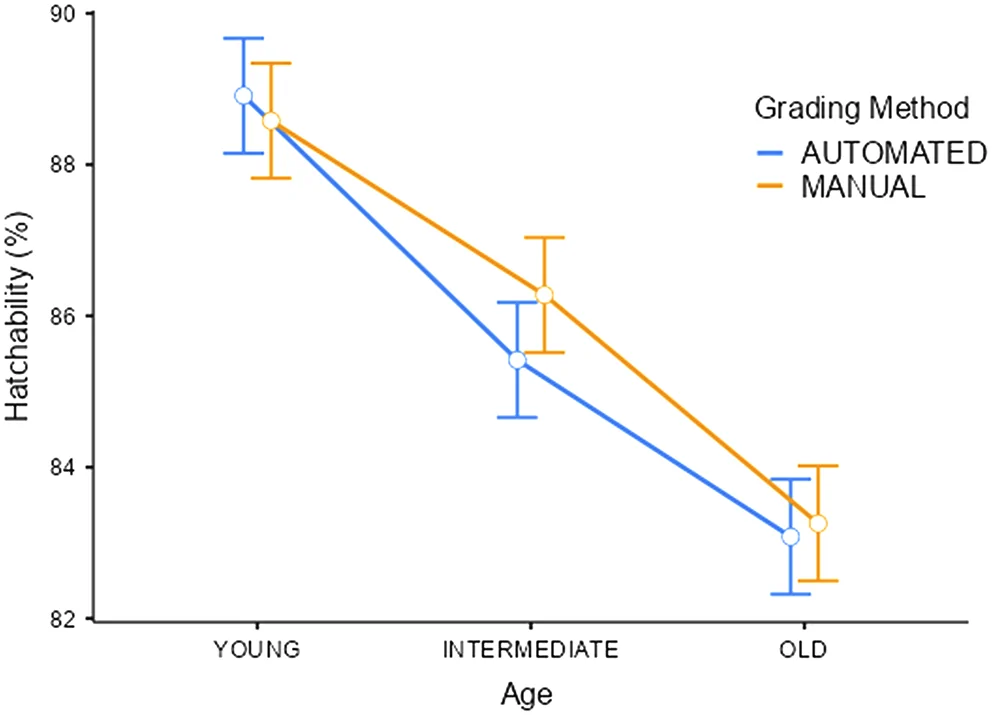
Hatchability rates according to breeder age and grading method. The figure illustrates the estimated marginal means for hatchability according to breeder age and grading method, with 95% confidence intervalss. Hatchability progressively decreased with advancing breeder age in both classification systems. Although automated classification showed slightly higher numerical hatchability values in younger flocks, the interaction between breeder age and grading method was not statistically significant, indicating that breeder age exerted a stronger influence on hatchability than the grading system itself. Source: Adapted from generalized linear model outputs generated in Jamovi from the experimental database

Figure 2 illustrates the interaction between breeder age and egg selection method on hatchability rate (95% confidence intervals). Although the interaction between breeder age and classification method was not statistically significant (*p* > 0.05), the figure demonstrates a progressive decline in hatchability with advancing breeder age across both classification systems. While the interaction did not reach statistical significance (*p* > 0.05), the graphical trend shows hatching rates declining with age across both methods a decline that appears more pronounced with manual selection. This pattern suggests a potential, though not statistically robust, combined effect between age and selection method during the transition from young to old matrices.

This graphical representation is crucial for uncovering nuances that significance tests may overlook, particularly in complex experiments. While the interaction lacks statistical significance, the diverging curves suggest that classification strategies should still be tailored to the breeder’s age. As breeder hens age, changes in egg composition directly influence embryonic mortality and hatch rates (Machado et al. 2020; Alo et al. 2024). These findings align with previous research linking bird aging to reduced eggshell quality, increased microcracks, and lower resistance to handling, all of which compromise overall productivity.

Manyeula et al. (2021) demonstrated that breeder age significantly impacts egg hatchability, noting that external and internal quality parameters such as weight, dimensions, and shell characteristics decline in Ross breeder hens between 30 and 60 weeks. Similarly, Rebolledo et al. (2023) reported that hatching rates for Ross 308 and Cobb 500 lines dropped from 85.34% at 30 weeks to 82.19% at 50 weeks. These findings suggest that advancing age reduces reproductive capacity and embryo viability, confirming that older breeder hens consistently produce eggs with lower incubation success.

In contrast to our findings, Salahi et al. (2012) reported that egg orientation significantly affects success, with inverted eggs showing a 4.3% reduction in hatchability due to compromised embryo respiration, dehydration, and membrane adhesion. Although previous studies have demonstrated that structural egg abnormalities may compromise hatchability, chick quality remains one of the primary indicators of incubation success. This aligns with Jabbar et al. (2019) and Perić et al. (2023), who noted that shell fragility in cracked eggs significantly lowers hatchability by increasing embryonic mortality during development.

The relationship between hen age and chick quality is a critical factor in the production of “second-rate” or “reject” chicks. Research by Franzo et al. (2020) and Adame and Ameha (2023) confirms that advanced breeder age leads to lower-quality eggs, resulting in higher embryonic mortality and increased reject rates. This decline is attributed to reduced reproductive efficiency, affecting both fertility and incubation capacity. Although the classification method did not significantly affect hatchability in the present study, breeder age consistently influenced incubation performance and chick quality across the evaluated flocks.

Statistical analysis indicates that while hen age moderately affects hatch rates particularly in older flocks the selection method alone lacks a statistically significant impact. However, hatchability is influenced by multiple biological and structural factors associated with breeder age and egg quality. Variations in shell integrity, egg composition, and embryonic viability may contribute to reductions in incubation efficiency as breeder hens age.

Incubation efficiency must extend beyond hatchability to include chick quality, as this parameter directly reflects the flock’s zootechnical potential. Our evaluation revealed that chick quality is influenced by the interaction between breeder age and classification method. Intermediate breeder hens produced the highest proportion of first-class chicks, benefiting from the optimal balance of shell strength and yolk nutrition (Oliveira and Santos 2018; Gregrova et al. 2024). In contrast, younger breeder hens produced chicks with lower vigor, while older breeder hens showed a higher incidence of second grade and reject chicks, likely due to shell fragility and size variability (Demirel and Demirel 2020; Schadek 2020). These findings suggest that subtle shell defects and structural irregularities may influence chick quality and embryo viability during incubation. These findings underscore that chick quality is a fundamental indicator of efficiency, defining both the volume and the long-term productive potential of the flock.

The decision to adopt automated grading systems should align with a facility’s production profile, technological capacity, and staff expertise. In large-scale units requiring high turnover and standardization, automation is a promising alternative that enhances operational efficiency and process traceability. However, successful implementation requires continuous calibration and sensor validation protocols tailored to the farm’s specific conditions, including age-related variations in the breeder hens. For older breeder hens which typically produce more fragile shells strategic nutritional supplementation is recommended. Incorporating organic minerals and highly bioavailable calcium can improve shell quality and reduce losses from cracks or breaks. This dietary support is essential to maximizing the accuracy of automated grading systems that rely on optical sensors or mechanical pressure.

## Conclusion

Breeder age significantly influenced hatchability outcomes, with progressive reductions observed as flock age advanced. In contrast, automated and manual grading systems showed comparable hatchability performance across the evaluated flocks, indicating that automation may be implemented without negatively affecting incubation efficiency. Although hatchability declined with advancing breeder age, both grading systems demonstrated consistent performance across different age groups.

Integrated strategies such as nutritional adjustments (calcium, phosphorus, and vitamin D), enhanced pre-incubation handling, and the development of more sensitive automated technologies—are essential to overcoming current grading limitations. With the growing shortage of skilled labor, automation has become a key driver for standardization and sustainability, though a hybrid approach with manual grading remains a strategic option for specific scenarios. Looking ahead, advancements in computer vision, AI, and non-invasive sensors are expected to provide highly accurate assessments of shell integrity and internal quality, supporting decision making in commercial hatcheries. Integrating these technologies into nutritional and reproductive management programs will maximize incubation efficiency and chick quality, ensuring a more productive and technically robust future for poultry farming.

## Data Availability

Not applicable.
